# Characteristics of the drinking habits of people who overdose over‐the‐counter drugs: Insights from a nationwide Japanese survey

**DOI:** 10.1002/pcn5.70027

**Published:** 2024-12-02

**Authors:** Satomi Mizuno, Satoshi Inoura, Toshihiko Matsumoto, Takuya Shimane

**Affiliations:** ^1^ Department of Drug Dependence Research, National Institute of Mental Health National Center of Neurology and Psychiatry Tokyo Japan; ^2^ Department of Nursing, Faculty of Nursing Niigata Seiryo University Niigata Japan

**Keywords:** energy drinks, illegal drugs, misuse, smoking, suicide

## Abstract

**Aim:**

To analyze the drinking habits of individuals who overdosed on over‐the‐counter (OTC) drugs, such as cough suppressants, antitussives, antipyretic analgesics, and combination cold medications, in Japan.

**Methods:**

This cross‐sectional study analyzed data collected from 2881 participants through a national survey conducted in Japan in 2023. Through a self‐administered questionnaire, the participants were categorized into OTC (overdosed OTC drugs in the past year) and control (did not overdose OTC drugs) groups. Variables related to drinking habits, social background, smoking habits, use of other medications, such as analgesics, tranquilizers, controlled drugs, and products containing high concentrations of caffeine, and perception of OTC drugs were compared between the two groups. Additionally, we matched the participants in the OTC (*n* = 25) and control (*n* = 100) groups by sex and age in a 1:4 ratio to assess these variables. Multivariate analyses were performed to examine how these factors are associated with drinking habits in individuals with OTC drug overdose.

**Results:**

OTC drug overdose was prevalent among teenagers and those in their 50s, and was associated with habitual binge drinking and consumption of energy drinks. Those overdosing on OTC drugs obtained them mostly from drugstores and pharmacies.

**Conclusion:**

This is the first study to show a relationship between drinking habits and OTC drug overdose. An understanding of the characteristics of drinking habits in those who overdose on OTC drugs can help reduce fatal health risks in this population.

## Graphical abstract

Table 2 shows the results of the multivariate logistic regression analyses before and after matching. Before matching, the model included variables that were significantly different: sex, age group, starting to smoke underage, habitual binge drinking, and habitual drinking of energy drinks. After matching, the model included habitual binge drinking and habitual drinking of energy drinks. Habitual alcohol consumption was excluded from the model before matching due to multicollinearity. The multivariable logistic regression analysis before matching revealed that over‐the‐counter (OTC) misuse was associated with the 10–19 and 50–59 years age groups compared to the 40–49 years age group (odds ratio [OR] 13.9, 95% confidence interval [CI] 1.2–310.5 and OR 14.1, 95% CI 2.8–258.5, respectively). OTC misuse was also linked to habitual binge drinking (OR 5.8, 95% CI 1.1–33.4) and habitual energy drink consumption (OR 23.8, 95% CI 3.4–476.4) in the multivariable logistic regression analysis after matching, similar to the results before matching.

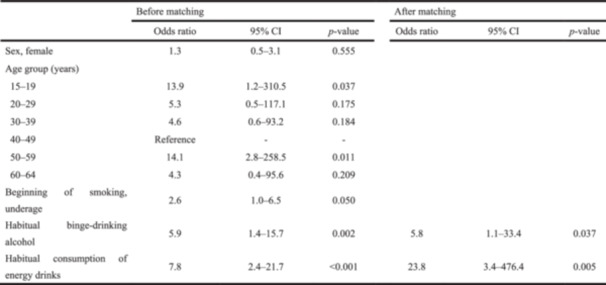

## INTRODUCTION

Globally, the number of people overdosing on over‐the‐counter (OTC) drugs, such as cough suppressants, antitussives, antipyretic analgesics, and general cold medicines, is increasing.^1^, ^2^, ^3^, ^4^, ^5^, ^6^ These medications contain psychoactive substances such as codeine,^7^ dextromethorphan,^8^, ^9^ diphenhydramine,^10^ ephedrine,^11^ and pseudoephedrine.^12^ These drugs are often misused by overdosing for non‐therapeutic purposes, mostly because OTC drugs are easily accessible and lack strict penalties, unlike controlled drugs. Overdosing OTC drugs is defined as the ingestion of large quantities of medicines containing psychoactive substances, such as cold or cough medicines, in a single instance, exceeding the recommended dosage, not for therapeutic purposes (e.g., relieving cold or cough symptoms) to change sensations or emotions. An overdose of OTC drugs can result in toxic or fatal blood levels, causing severe health problems or death.^3^, ^13^ For example, an overdose of OTC drugs containing acetaminophen can cause liver damage,^14^ and an overdose of drugs containing codeine or dextromethorphan can lead to poisoning.^2^, ^3^, ^15^, ^16^ There are reports of people committing suicide by overdosing on OTC drugs.^17^, ^18^, ^19^, ^20^, ^21^

The risks associated with OTC drug overdosing are further increased by alcohol consumption.^22^, ^23^, ^24^ Postmortem examination of individuals who died from OTC drug overdoses, especially those who committed suicide, often show the presence of alcohol in their bodies.^25^, ^26^ Alcohol consumption along with an OTC drug overdose accelerates the absorption of the drug's active ingredients, leading to a rapid increase in its blood levels, providing a faster euphoric experience, but also increasing the risk of poisoning, which can be fatal.^27^ Therefore, even if the OTC drugs are within the recommended therapeutic doses,^28^, ^29^ concomitant drinking alcohol should not be allowed due to increased fatal health risks.

Fatal health risks associated with OTC drug overdoses are well known. However, the characteristics of drinking habits in people who overdose on OTC drugs are not well understood. While previous studies have primarily focused on demographic characteristics, such as the age and sex of those who overdose on OTC drugs, they did not investigate the drinking habits of these individuals.^1^, ^30^, ^31^, ^32^, ^33^, ^34^, ^35^, ^36^ These studies found a higher proportion of women and younger individuals with mental disorders overdosing on OTC drugs.^1^, ^30^, ^31^, ^33^, ^34^, ^36^ Many patients who repeatedly overdose on these drugs develop dependence and experience severe withdrawal symptoms similar to opioid addiction, making self‐control difficult.^32^ In Japan, the number of patients with OTC drug use disorders in psychiatric hospitals and emergency cases related to OTC drug overdose has been increasing.^30^, ^33^, ^34^, ^36^ To reduce the health risks and fatal outcomes, it is crucial to understand the characteristics of drinking habits in people who overdose on OTC drugs. Additionally, many factors have been reported to be associated with alcohol consumption. Among them, smoking and the use of medicines, controlled drugs, and products containing high concentrations of caffeine are known to affect drug metabolism. Therefore, when combined with alcohol consumption, these factors may potentially cause adverse health effects in individuals who overdose on OTC drugs.

This study mainly aimed to analyze the drinking habits of individuals who overdose on OTC drugs. Additionally, we examined the effects of smoking, and the consumption of controlled drugs and high caffeine‐containing products on OTC drug overdosing. These factors are known to be associated with drinking habits and negatively affect drug metabolism. Data on these factors were collected from a 2023 national survey in Japan.^35^ We compared the people who overdosed on OTC drugs with those who did not. Additionally, we examined the sources of the OTC drugs used to overdose by those who overdosed on OTC drugs.

## METHODS

### Design

This cross‐sectional study used a self‐administered questionnaire and focused on individuals who had overdosed (taken more than the prescribed dose at a time) on OTC drugs containing psychoactive substances, such as cough medicines, cold medicines, and antipyretic analgesics, for no therapeutic purposes (e.g., relieving cold or cough symptoms), but to alter sensations or emotions. Data were collected from the Nationwide General Population Survey on Drugs,^35^ a national survey conducted in 2023. The study period was from October 16 to December 22, 2023.

### Participant enrolment

Participants included 5000 individuals aged 15–64 years selected from 250 locations across Japan using a stratified two‐stage random sampling method.^35^ Japan's prefectures were divided into 11 districts, which were further divided into 65 extractive unit areas (blocks) based on city size (Table S1). City size was classified based on the population recorded in the Basic Resident Register on January 1, 2022. The number of survey cities in each block was determined to avoid bias, with 13–23 participants sampled from each city proportionally distributed according to the population density.

In the first phase, cities were randomly selected using a random number table, ensuring equal sampling intervals by dividing the population aged 15–64 years in each block by the number of cities surveyed. The order of the municipalities followed that of the 2020 census.

In the second phase, participants were randomly selected from each survey city using the Basic Resident Register. The sampling interval was calculated by dividing the population aged 15–64 years in each survey city by the number of cities sampled within each block. This method ensured a nationally representative sample by minimizing regional and population density biases.

### The nationwide general population survey

Survey documents (study description, questionnaire form, return envelope, and Internet response guide) were mailed to 5000 participants. Participants responded using either a paper questionnaire or an Internet survey. Consent was confirmed by completing the questionnaire or checking the “I agree” box on the online consent form. Paper respondents mailed the completed questionnaires in the provided envelope. The Internet Response Guide included a login ID, password, response procedure, QR code, and URL for the survey. Participants could respond via computers, smartphones, or tablets and submit their responses once all questions were answered.

### Ethical considerations

The study protocol was reviewed and approved by the Ethics Committee of the National Center of Neurology and Psychiatry in Japan (A2023‐031). The survey did not require informed consent. Instead, an opt‐out approach was used. Participants were informed about the survey through a public notice approved by the Ethics Committee and posted on the website. This notice guaranteed the right to refuse participation and withdraw from the survey at any time, even after submitting the completed questionnaires. The withdrawal procedure was detailed in the public notice, survey descriptions, and questionnaire forms. These documents stated: “Responses are anonymous,” “Participation is voluntary,” and “There are no disadvantages for not participating.” This study adhered to the principles of the Declaration of Helsinki.

### Participant selection

We excluded 1974/5000 participants surveyed nationwide in the 2023 General Population Survey on Drug Use. The proportion of the number of participants at each location to the total study population (*n* = 5000) was 0.08%–0.24% and was comparable to the proportion of the number of respondents at each location to the total respondents in this study (*n* = 3026).

**Figure 1:**
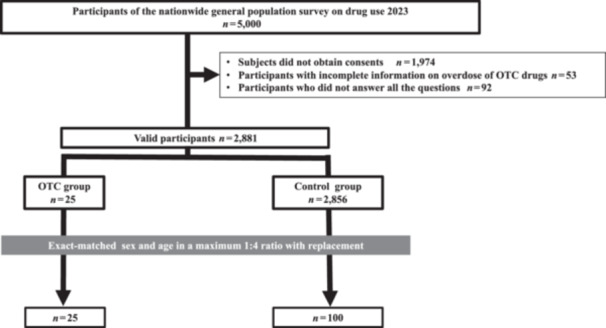
Flowchart of participant selection and the matching process. Through a self‐administered questionnaire, the participants were asked, “In the past year, have you overdosed on OTC cough medicines, cold medicines, or fever reducers for abusive (not therapeutic) purposes? Abusive use was defined as the use of more than a set amount or number of times to get high or change mood.” Those who answered “yes” were classified into the OTC drug overdose group. Those who answered “no” were classified into the control group. OTC, over‐the‐counter.

As our study focused on individuals engaged in OTC drug overdose, we excluded 53 participants with incomplete information regarding the overdose and 92 who did not complete all the survey questions. Finally, we included 2881 participants in the study. Based on their responses to a self‐administered questionnaire on OTC drug overdose within the past year, the participants were categorized into OTC (participants who overdosed on OTC drugs, *n* = 25) and control (participants who did not overdose on OTC drugs, *n* = 2856) groups as shown in Figure 1.

### Variables

The OTC and control groups were compared for several variables, including sex, age (mean), age group (in 10‐year increments), employment status, educational level, drinking habits, smoking habits, use of high caffeine‐containing products, medicines, and controlled drugs, and the perception of OTC overdose. These variables were selected based on previous reports that focused on patients with OTC drug use disorders in psychiatric hospitals and emergency cases.^30^, ^33^, ^34^, ^36^

In addition to the average age, we included the number of people in each age group as a variable because while the average age shows a central tendency, it does not capture the spread or bias of the age distribution. A comparison of the number of individuals in each age group helped identify potential biases. We also categorized employment status into self‐employed, full‐time, part‐time, student, other occupations, and unemployed.

The drinking habits were categorized into (a) started drinking before 20 years of age, (b) drinking for the last month, (c) habitual drinking for the last year, and (d) habitual binge drinking for the last year. Based on the National Health and Nutrition Survey, habitual drinking was defined as drinking alcohol for more than 3 days every week for a month, while habitual binge drinking was drinking more than approximately 1400 ml of alcohol with an alcohol content of 5% or more on a single occasion, more than 3 days a week for a month.^37^ In this study, we categorized drinking habit variables in several ways because different patterns of drinking habits (such as the age of initiation, frequency of drinking, and quantity consumed) are associated with different health risks and behaviors. Each of these patterns may have a different impact on OTC drug overdose. This approach aimed to evaluate how each drinking habit specifically influences OTC drug overdose.

The smoking habits were categorized as started smoking before 20 years of age (underage) and heavy smoking. Based on the National Health and Nutrition Survey, heavy smoking was defined as smoking for more than 20 days per month.^37^

The status of consumption of high‐caffeine products, such as energy drinks and caffeine tablets, was added as a new variable. Overdosing with caffeine‐containing OTC drugs can lead to caffeine poisoning if consumed simultaneously with high‐caffeine products. Habitual intake of these products was defined as consumption three or more times a week.

Considering that people who overdose on OTC drugs tend to have mental disorders, we added the habitual use of analgesics and tranquilizers as another variable. People with chronic pain are more likely to have mental disorders.^38^, ^39^, ^40^ The habitual use of analgesics and tranquilizers in combination with OTC drug overdosing was added as a variable because of its adverse effects. Habitual use of these medications was defined as their use at least three times per week in the past year.

We included variables related to the use of controlled drugs such as marijuana, methamphetamine, inhalants, MDMA, cocaine, heroin, new psychotropic substances, and lysergic acid diethylamide in the past year. In addition, thoughts on the acceptability of controlled drug use, specifically marijuana, which is the most commonly used controlled drug in Japan, were also added as a variable.^35^, ^41^, ^42^, ^43^ This variable was added because we believe that people overdosing on OTC drugs might have different ideas about using them than those who did not misuse them.

Finally, we added variables to assess participants’ knowledge of the health risks associated with OTC drug overdose. These included awareness that overdosing on OTC drugs can lead to addiction and that taking large doses can cause death.^44^, ^45^

### Statistical analyses

We compared the characteristics of the OTC and control groups using Student's *t*‐tests for continuous variables and chi‐square tests for categorical variables. The participants in the OTC and control groups were exact‐matched by age and sex in a 1:4 ratio to control for differences that could distort the relationship between OTC overdose and other variables, including drinking habits and smoking habits.

Using multivariable logistic regression analyses, we quantified the impact of each variable and calculated its odds ratios. We checked the variance inflation factors to examine multicollinearity, and factors <10 indicated no multicollinearity. All statistical analyses were performed using R version 4.2.3 (R Foundation for Statistical Computing, Vienna, Austria) (https://www.r-project.org/). Statistical significance was set at P < 0.05. A multivariate regression analysis was performed before matching, and the 40–49 age group was used as the reference. Since a previous study showed a higher proportion of OTC drug use disorder in teens and those in their 20s compared to those in their 30s and 40s,^36^ we evaluated the relationship with each age group using 40s as the reference.

For the OTC group, we further investigated the sources of OTC drugs used for overdose, classifying them as pharmacies and drugstores, the Internet, transfer from an acquaintance or family member, regular home medicine, other sources, or unknown sources.

## RESULTS

The sex, age, and age group trends of the participants are shown in Table S2. Before matching, the mean (standard deviation) ages of the OTC and control groups were 46.8 (14.3) and 43.4 (13.7) years, respectively (P = 0.221). There were no sex‐based differences between the two groups (44.0% vs. 50.6%, P = 0.649) (Table 1). However, significant differences were seen in age before matching (P = 0.015). Compared to the control group, the OTC group had higher proportions of people aged 15–19 (8.0% vs. 6.5%) and 50–59 (60.0% vs. 28.1%). After matching, the variables significantly associated with OTC overdose were habitual binge drinking and habitual energy drink consumption.

**Table 1:** Comparison of the characteristics between the OTC and control groups before and after matching.

	Before matching			After matching		
	OTC group (*n* = 25)	Control group (*n* = 2856)	P value	OTC group (*n* = 25)	Control group (*n* = 100)	P value
Variables used for matching						
Sex, female, number (%)	11 (44.0)	1,445 (50.6)	0.649	11 (44.0)	44 (44.0)	1.000
Age in years (mean (SD))	46.8 (14.3)	43.4 (13.7)	0.221	46.8 (14.3)	46.8 (14.1)	1.000
Age group (years)			0.015			1.000
15–19	2 (8.0)	185 (6.5)		2 (8.0)	8 (8.0)	
20–29	2 (8.0)	364 (12.7)		2 (8.0)	8 (8.0)	
30–39	3 (12.0)	498 (17.4)		3 (12.0)	12 (12.0)	
40–49	1 (4.0)	650 (22.8)		1 (4.0)	4 (4.0)	
50–59	15 (60.0)	802 (28.1)		15 (60.0)	60 (60.0)	
60–64	2 (8.0)	357 (12.5)		2 (8.0)	8 (8.0)	
Variables not used for matching						
Employment status						
Unemployed	1 (4.0)	144 (5.0)	1.000	1 (4.0)	4 (4.0)	1.000
Self‐employed	3 (12.0)	218 (7.6)	0.660	3 (12.0)	5 (5.0)	0.411
Full‐time	11 (44.0)	1506 (52.7)	0.503	11 (44.0)	59 (59.0)	0.260
Part‐time	5 (20.0)	377 (13.2)	0.483	5 (20.0)	12 (12.0)	0.473
Student	3 (12.0)	254 (8.9)	0.849	3 (12.0)	9 (9.0)	0.939
Other work	2 (8.0)	357 (12.5)	0.708	2 (8.0)	11 (11.0)	0.942
High‐school graduate	12 (48.0)	1818 (63.7)	0.158	12 (48.0)	63 (63.0)	0.254
Drinking alcohol status						
Beginning, underage	16 (64.0)	1557 (54.5)	0.455	16 (64.0)	68 (68.0)	0.886
Drinking in the past month	18 (72.0)	1722 (60.3)	0.324	18 (72.0)	60 (60.0)	0.380
Habitual drinking	11 (44.0)	542 (19.0)	0.004	11 (44.0)	27 (27.0)	0.159
Habitual binge drinking	4 (16.0)	74 (2.6)	<0.001	4 (16.0)	3 (3.0)	0.041
Smoking status						
Beginning, underage	14 (56.0)	894 (31.3)	0.015	14 (56.0)	37 (37.0)	0.133
Heavy smoker	5 (20.0)	545 (19.1)	1.000	5 (20.0)	33 (33.0)	0.307
Status of taking the products, including high levels of caffeine						
Habitual drinking of energy drinks	5 (20.0)	115 (4.0)	0.001	5 (20.0)	1 (1.0)	0.001
Habitual use of caffeine tablets	1 (4.0)	74 (2.6)	1.000	1 (4.0)	1 (1.0)	0.859
Status of using the medications for therapeutic purposes						
Habitual use of analgesics	1 (4.0)	103 (3.6)	1.000	1 (4.0)	3 (3.0)	1.000
Habitual use of tranquilizers	2 (8.0)	114 (4.0)	0.614	2 (8.0)	3 (3.0)	0.568
Have used controlled drugs	0 (0.0)	90 (3.2)	0.746	0 (0.0)	6 (6.0)	0.464
Perception about using controlled drugs						
Marijuana should not be used	24 (96.0)	2769 (97.0)	1.000	24 (96.0)	96 (96.0)	1.000
Knowing that abusing OTC can lead to addiction	6 (24.0)	628 (22.0)	1.000	6 (24.0)	23 (23.0)	1.000
Knowing that taking large doses of OTC at once can cause death	3 (12.0)	369 (12.9)	1.000	3 (12.0)	14 (14.0)	1.000

Abbreviations: OTC, over‐the‐counter; SD, standard deviation.

*Note*: Data are presented as *n* (%) unless otherwise indicated. Drinking, beginning underage: Started drinking before the age of 20 years. Drank in the past month: Drank alcohol within the past month. Habitual drinking: Drank alcohol more than three times every week. Habitual binge drinking: Engaged in binge drinking, defined as consuming more than approximately 1400 ml of alcohol with an alcohol content of 5% or more on a single occasion. Smoking, beginning underage: Started smoking before the age of 20. Heavy smoking: Smoking more than 20 days per month. Habitual drinking of energy drinks: Drank energy drinks more than 20 days per month. Habitual use of caffeine tablets: Took caffeine tablets for over 20 days per month. Habitual use of analgesics: Took analgesics more than three times every week. Habitual use of tranquilizers: Took tranquilizers more than three times every week. We considered P < 0.05 to indicate statistical significance.

Table 2 shows the results of the multivariate logistic regression analyses before and after matching. Before matching, the model included variables that were significantly different: sex, age group, starting to smoke underage, habitual binge drinking, and habitual drinking of energy drinks. After matching, the model included habitual binge drinking and habitual drinking of energy drinks. Habitual alcohol consumption was excluded from the model before matching due to multicollinearity. The multivariable logistic regression analysis before matching revealed that OTC overdose was associated with the 15–19 and 50–59 years age groups compared to the 40–49 years age group (odds ratio [OR] 13.9, 95% confidence interval [CI] 1.2–310.5 and OR 14.1, 95% CI 2.8–258.5, respectively). OTC overdose was also linked to habitual binge drinking (OR 5.8, 95% CI 1.1–33.4) and habitual energy drink consumption (OR 23.8, 95% CI 3.4–476.4) in the multivariable logistic regression analysis after matching, similar to the results before matching.

**Table 2:** Multivariable conditional logistic regression analysis.

	Before matching			After matching		
	Odds ratio	95% CI	P value	Odds ratio	95% CI	P value
Sex, female	1.3	0.5–3.1	0.555			
Age group (years)						
15–19	13.9	1.2–310.5	0.037			
20–29	5.3	0.5–117.1	0.175			
30–39	4.6	0.6–93.2	0.184			
40–49	Reference	‐	‐			
50–59	14.1	2.8–258.5	0.011			
60–64	4.3	0.4–95.6	0.209			
Beginning of smoking, underage	2.6	1.0–6.5	0.050			
Habitual binge‐drinking alcohol	5.9	1.4–15.7	0.002	5.8	1.1–33.4	0.037
Habitual consumption of energy drinks	7.8	2.4–21.7	<0.001	23.8	3.4–476.4	0.005

Abbreviation: CI, confidence interval.

*Note*: Habitual binge drinking alcohol: Engaged in binge drinking, defined as consuming more than approximately 1400 ml of alcohol with an alcohol content of 5% or more on a single occasion. Beginning of smoking, underage: Started smoking before the age of 20. Habitual drinking of energy drinks: Participants drank energy drinks more than 20 days per month. Statistical significance was set at P < 0.05. The Akaike's information criteria for the model before and after matching were 270.81 and 115.11, respectively. The variables that were significantly associated with OTC overdose before matching were habitual binge drinking, smoking, and habitual energy drink consumption. These variables were entered into a logistic regression model. The variables that were significantly associated with OTC overdose after matching were habitual alcohol binge drinking and habitual energy drink consumption, therefore these two variables were entered into a logistic regression analysis model.

The sources of OTC drugs used for overdose were physical shops, such as pharmacies and drugstores (36%), regular medicines at home (16%), the Internet (4%), and unknown sources (56%).

## DISCUSSION

To the best of our knowledge, this study is the first to use the National General Population Survey to compare the characteristics of drinking habits of people who overdosed on OTC drugs with those who did not. This study found that OTC drug overdose was particularly prevalent among teenagers and middle‐aged adults, and was associated with habitual binge drinking. Additionally, the habitual use of high‐caffeine products such as energy drinks has also been linked to OTC drug overdose. One‐third of those who overdosed on OTC drugs obtained them from pharmacies or drugstores.

### Age groups

We found that people who overdosed on OTC drugs were predominantly teenagers, which is consistent with previous studies showing that many psychiatric patients addicted to OTC drugs are teenagers.^46^ This study also found that people in their 50s frequently overdosed on OTC drugs. As with teenagers, individuals in their 50s who overdose on OTC drugs may also suffer from mental illnesses, such as depression. In addition, in teenagers, overdosing is associated with loneliness, stress, and disrupted habits such as poor sleep and skipping breakfast.^1^, ^47^, ^48^ Therefore, it is likely that middle‐aged and older adults who overdose on OTC drugs may also be facing stress and loneliness.

This study found no significant differences in the sex of participants who overdosed on OTC drugs. This is in contrast to previous surveys that showed more women overdosing on OTC drugs.^36^, ^49^ This discrepancy could be due to our study's broader age range (15–64 years) and general population survey, while previous studies focused on high school students^49^ and psychiatric hospital patients,^36^ leading to different participant backgrounds. Since this study only included participants aged 15 and above, the exclusion of those under 15, who were included in previous studies, might have also impacted the results. Additionally, the sample size for young women was limited, which may have made it difficult to detect significant sex‐based differences in risk. Based on this, future research using a larger dataset, including a more targeted sample of young women, is needed to examine sex‐based differences in risk more thoroughly.

### Binge‐drinking alcohol

Our results show that individuals who overdosed on OTC drugs tended to engage in heavy drinking habits. Previous studies have suggested that those who overdose on OTC drugs in combination with alcohol are at a significant risk of fatal health issues.^22^, ^23^, ^24^, ^28^, ^50^ Additionally, there have been reports of individuals intentionally consuming alcohol to enhance the effects of OTC drugs when attempting suicide.^29^, ^51^, ^52^, ^53^, ^54^ People who overdose on OTC drugs and engage in heavy drinking might be trying to cope with mental distress and stress. Another reason could be to commit suicide. Drinking alcohol, self‐harming, and OTC drug overdoses are temporary methods to relieve stress and mental pain.^55^ Moreover, a previous study suggested a close relationship between substance use, including OTC drug overdose, binge alcohol consumption, and suicidal ideation, suggesting that these substances alleviate feelings of loneliness or hopelessness.^56^ Additionally, some people binge on drinking alcohol and overdose on OTC drugs as alternatives to self‐harm or to alleviate suicidal thoughts.^19^, ^53^ Although these agents offer temporary relief, they ultimately do more harm.^50^

The age groups associated with OTC drug overdose in this study (teenagers and those in their 50s) were more likely to be engaged in habitual binge drinking, suggesting that they may also be at a very high risk of fatal outcomes, including suicide. The age groups with the highest suicide rates in Japan are 15–24 and 40–59 years,^57^ which aligns with the findings of our study. Individuals who overdose on OTC drugs experience mental health issues such as depression and anxiety, which can lead to suicidal ideation and create a vicious cycle of OTC drug overdose. Based on these findings, psychological support, including suicide prevention measures, may be necessary not only for young people but also for middle‐aged individuals who overdose on OTC drugs. However, this study did not evaluate the mental state of the participants, therefore further research is needed to investigate the relationship between OTC drug overdose, drinking habits, and suicide.

### Consumption of energy drinks

Consistent with previous studies focusing on controlled and prescription drugs,^58^, ^59^, ^60^, ^61^ we found a significant association between OTC drug overdose and energy drink consumption. Caffeine from energy drinks combined with an overdose of OTC drugs, including caffeine, can lead to coronary artery spasms, thrombosis, cardiac arrhythmias, and sudden cardiac death.^59^ It is therefore crucial to warn individuals who overdose on OTC drugs of the fatal health risks associated with these substances.

OTC drug overdosing is often combined with the consumption of energy drinks to counter intoxication by drinking or to enhance the drug's effects. A significant relationship between drinking habits and the habitual consumption of energy drinks has been previously reported.^60^, ^61^, ^62^ In addition, combining caffeine and alcohol reduces the perceived intoxication, increases stimulation, and heightens the desire to consume more alcohol, thereby increasing the associated risks.^63^ Mixing energy drinks with alcohol is linked to a higher risk of binge drinking, impaired driving, risky sexual behavior, and alcohol dependence than consuming alcohol alone.^62^, ^63^, ^64^, ^65^ We found that OTC drug overdose was significantly associated with the consumption of energy drinks but not caffeine tablets. The finding might be that energy drinks are readily available at convenience stores and vending machines, whereas caffeine tablets are sold only at pharmacies for limited hours.

To the best of our knowledge, this is the first study to report a direct relationship between energy drinks and OTC drug overdose. While several studies have previously suggested a link between energy drinks and the use of illicit drugs or prescription medications,^58^, ^66^, ^67^ these analyses typically adjusted for factors such as sex, age, and race, without specifically considering the impact of alcohol consumption. In contrast, we adjusted for both alcohol and energy drink consumption, and the results suggest a potential association between energy drinks and OTC drug overdose. However, it is important to acknowledge that other unknown variables may not have been fully accounted for, and the possibility remains that factors other than alcohol may still be influencing this relationship.

### Sources of OTC drugs used for overdose

We found that people who overdose on OTC drugs are more likely to obtain them from physical stores, such as drugstores and pharmacies, rather than online. Our results align with previous studies showing that consumers prefer physical stores for OTC drug purchases, often due to face‐to‐face consultations with pharmacists.^66^, ^67^, ^68^, ^69^ Most consumers in countries such as Sweden^68^ (76%) and Japan^67^ (89%) still prefer buying OTC drugs from physical stores to online. While the one‐third rate in our study is lower than the result of previous studies, it is still significantly higher than the 4% of misusers who purchase online.

However, the reasons to choose physical shops over online in general consumers may differ from people who overdose on OTC drugs. People who overdose on OTC drugs may choose physical stores for quick access to drugs, which is harder to achieve with online purchases.

As online purchasing increases among convenience‐seeking consumers,^67^ and with some OTC drugs legally obtainable online in Japan, there might be a growing need for policy discussions on how interventions for OTC drug overdose should be implemented in physical stores and online purchases. Our findings might suggest that in‐store interventions involving pharmacists as gatekeepers could play a critical role in preventing OTC drug overdose. Some countries, including Japan, are already working to establish pharmacists in this preventative role.^70^, ^71^, ^72^, ^73^

### Limitations

This study has some limitations. First, using a self‐administered questionnaire for the general population requires keeping the questions simple and easy to understand, and omitting detailed questions about the ingredients and product names of the OTC drugs. Therefore, we could not investigate the characteristics of individuals who overdosed on OTC drugs based on different ingredients or products. Second, the data were collected through self‐reporting, which may have introduced reporting bias. Third, the mental health status of the participants was not assessed, which may have affected the OTC drug overdose. Fourth, the frequency and amount of overdose of OTC drugs were not evaluated, which could have influenced the findings of the study.

The primary objective of this study was to evaluate potential associations between OTC drug overdosing and drinking habits and to explore how other factors, such as smoking, consumption of high‐caffeine products, and use of medicine and controlled drugs, might influence this relationship. Since there are several common causes for drinking and OTC drug overdose, it is difficult for a cross‐sectional study like this one to clarify the causal relationship between the two. However, the findings from this study could serve as a foundation for future longitudinal studies to further investigate causality.

## CONCLUSION

Our results showed that the overdose of OTC drugs was more common among those in their teens and 50s and was significantly associated with habitual binge drinking. OTC drug overdosing was also associated with the consumption of high‐caffeine products, such as energy drinks. Finally, overdosers mostly obtain OTC drugs from drugstores and pharmacies. We believe our findings can help in reducing the health risks and fatal outcomes of OTC drug overdose by addressing the associated factors and regulating the availability of these drugs to populations who are likely to overdose on them.

## AUTHOR CONTRIBUTIONS

**T.S.** and **S.M.** designed the preliminary experiments. **S.I.** and **S.M.** recruited participants and collected data. **T.S**., **S.I**., and **S.M.** established the database of research participants. **T.S.** obtained funding. **S.M.** designed the study, performed statistical analyses, and wrote the manuscript. **T.S.**, **S.I.**, and **T.M.** supervised the study design and wrote the manuscript. S.M. wrote the initial draft of the manuscript. All authors revised and contributed to the final version of the manuscript. All authors have read and approved the final manuscript for publication.

## CONFLICT OF INTEREST STATEMENT

The authors declare no conflict of interest.

## ETHICS APPROVAL STATEMENT

The study protocol was reviewed and approved by the Ethics Committee of the National Center of Neurology and Psychiatry of Japan (A2023‐031) and conformed to the provisions of the Declaration of Helsinki.

## PATIENT CONSENT STATEMENT

Informed consent was not obtained for the survey. Instead, an opt‐out approach was adopted. The participants could withdraw from the research at any time during the study. The information on how to cancel their participation was posted on the website of the Ethics Committee of the institution concerned.

## CLINICAL TRIAL REGISTRATION

N/A.

## Supporting information


**SUPPORTING INFORMATION TABLE S1** Number of participants and survey cities in each block of Japan.
**SUPPORTING INFORMATION TABLE S2** The sex, age, and age group tendencies in the entire cohort.

## Data Availability

In order to protect the confidentiality of study participants, the data are unavailable.
