# Supplementary material for: Characteristics of the drinking habits of people who overdose over‐the‐counter drugs: Insights from a nationwide Japanese survey
Source: PCN Rep. 2024 Dec 2;3(4):e70027. doi: 10.1002/pcn5.70027 (PMC11612021; doi:10.1002/pcn5.70027)
Supplement: Supplementary file 1 — SUPPORTING INFORMATION TABLE S1 Number of participants and survey cities in each block of Japan. SUPPORTING INFORMATION TABLE S2 The sex, age, and age group tendencies in the entire cohort. [file PCN5-3-e70027-s001.docx]

**Supporting information**

**Supplementary Table 1**. **Number of participants and survey cities in each block of Japan.**

| Regions | Metropolis | | | | | | Cities with a population≥ 200,000 | Cities with a population ≥ 100,000 | Cities with a population < 100,000 | County (Town/Village) | Total |
| --- | --- | --- | --- | --- | --- | --- | --- | --- | --- | --- | --- |
|  | Tokyo’s 23 wards | Yokohama City | Kawasaki and  Kyoto Cities | Chiba , Nagoya , and Osaka Cities | Saitama , Shizuoka, Kobe , Hiroshima , and Kitakyushu cities | Other metropolises |  |  |  |  |  |
| Hokkaido |  |  |  |  |  | 80 | 21 | 32 | 33 | 32 | 198 |
|  |  |  |  |  |  | (4) | (1) | (2) | (2) | (2) | (11) |
| Tohoku |  |  |  |  |  | 45 | 87 | 33 | 105 | 53 | 323 |
|  |  |  |  |  |  | (2) | (4) | (2) | (5) | (3) | (16) |
| Kanto | 427 | 159 | 68 | 41 | 57 | 30 | 446 | 298 | 220 | 69 | 1,815 |
|  | (19) | (7) | (3) | (2) | (3) | (2) | (20) | (14) | (10) | (4) | (84) |
| Hokuriku |  |  |  |  |  | 30 | 54 | 22 | 73 | 14 | 193 |
|  |  |  |  |  |  | (2) | (3) | (1) | (4) | (1) | (11) |
| Tousan |  |  |  |  |  |  | 40 | 33 | 82 | 31 | 186 |
|  |  |  |  |  |  |  | (2) | (2) | (4) | (2) | (10) |
| Tokai |  |  |  | 96 | 27 | 31 | 110 | 119 | 106 | 33 | 522 |
|  |  |  |  | (5) | (2) | (2) | (5) | (6) | (5) | (2) | (27) |
| Kinki |  |  | 56 | 117 | 60 | 33 | 253 | 103 | 152 | 40 | 814 |
|  |  |  | (3) | (6) | (3) | (2) | (12) | (5) | (7) | (2) | (40) |
| Chugoku |  |  |  |  | 48 | 28 | 53 | 71 | 52 | 19 | 271 |
|  |  |  |  |  | (3) | (2) | (3) | (4) | (3) | (1) | (16) |
| Shikoku |  |  |  |  |  |  | 59 | 18 | 40 | 20 | 137 |
|  |  |  |  |  |  |  | (3) | (1) | (2) | (1) | (7) |
| Northern Kyushu |  |  |  |  | 35 | 67 | 63 | 39 | 80 | 35 | 319 |
|  |  |  |  |  | (2) | (3) | (3) | (2) | (4) | (2) | (16) |
| Southern Kyushu |  |  |  |  |  | 29 | 52 | 43 | 61 | 37 | 222 |
|  |  |  |  |  |  | (2) | (3) | (2) | (3) | (2) | (12) |
| Total | 427 | 159 | 124 | 254 | 227 | 373 | 1,238 | 811 | 1,004 | 383 | 5,000 |
|  | (19) | (7) | (6) | (13) | (13) | (21) | (59) | (41) | (49) | (22) | (250) |

Upper row: number of participants; lower row: number of survey cities.

**Supplementary Table 2. The sex, age, and age group tendencies in the entire cohort**

|  | Total  (n = 2,881) |
| --- | --- |
| **Variables used for matching:** |  |
| Female | 1,425(49.5) |
| Age (mean [SD] years) | 43.5[13.7] |
| Age-group (years old) |  |
| 15-19 | 187(6.5) |
| 20-29 | 366(12.7) |
| 30-39 | 501(17.4) |
| 40-49 | 651(22.6) |
| 50-59 | 817(28.4) |
| 60-64 | 359(12.5) |

Data are presented as n (%), unless otherwise indicated.

Abbreviations: SD, standard deviation.
